# Progression of ascending aortic dilatation in congenital bicuspid aortic valve as characterised by cardiovascular magnetic resonance

**DOI:** 10.1186/1532-429X-15-S1-E87

**Published:** 2013-01-30

**Authors:** Lay Koon Tan, Yumi Shiina, Beatrice Bonello, Raad H Mohiaddin

**Affiliations:** 1 CMR, Royal Brompton Hospital, London, UK; 2 National Heart and Lung Institute, Imperial College, London, UK; 3 Cardiology, National Heart Institute, Kuala Lumpur, Malaysia

## Background

Although bicuspid aortic valve (BAV) is the commonest congenital cardiac defect, longitudinal studies investigating the natural history of its associated aortic dilatation are scarce. We aim to retrospectively investigate the progression of ascending aortic (Asc Ao) dilatation in BAV patients.

## Methods

A retrospective analysis of serial Asc Ao dimension was made of 31 patients (mean age 46±14, 68% male and 32% female) with BAV who underwent at least 2 cardiovascular magnetic resonance imaging study (CMR) > 4 months apart from January 2002 to September 2012 at the Royal Brompton Hospital. Patients with associated aortic coarctation or aortic valvular dysfunction were excluded. Images were acquired with either a Siemens Avanto or Siemens Sonata scanner. Maximal intraluminal Asc Ao diameters at the level of right pulmonary artery (RPA) were measured in end diastole using the axial single-shot spin-echo (HASTE) sequence. The rate of change in aortic dimension per year was calculated.

## Results

There were 19 BAV patients with baseline Asc Ao dimension of <40 mm (Group A) and 12 BAV patients with ≥40 mm (Group B). The mean baseline Asc Ao diameters in group A was 32±3.4 mm (indexed 17±2.4 mm/m^2^) and 47.3±4.4 mm (indexed 24.3±2 mm/m^2^). The average time between CMR studies was 28.2±19 months (4 to 80.4 months). The annual rate of progression of Asc Ao dilatation was 0.6±0.72 mm/year in group A and 0.5±0.3 4 mm/year in group B, p=0.2.

## Conclusions

The annual progression rate of Asc Ao dilatation in BAV patients with baseline dimension of <40 mm was 0.6mm±0.72 mm/year while those with ≥40 mm was 0.5±0.34 mm/year. The difference in progression between these 2 groups of BAV patients are not statistically significant and are comparable with previous longitudinal studies [Thanassoulis et al., Nat Clin Prac Cardio Med, 2008 V5, http://www.nature.com/clinicalpractice/cardio].

## Funding

No Funding

